# American Medical Association

**Published:** 1853-06

**Authors:** 


### REPORTS OF SOCIETIES.

### AMERICAN MEDICAL ASSOCIATION.

*Held at New York, May 3d*, 1853.

The American Medical Association held their sixth annual meeting in the Presbyterian Church, Bleecker street, the President, Dr. BEVERLY R. WELLFORD, of Va. in the Chair. The morning was occupied by the Committee of Arrangements, in receiving delegates from the several States. At 11½ A. M., the meeting organized, and the President welcomed the Delegates to the City. There were nearly five-hundred gentlemen present.

Dr. F. CAMPBELL STEWART, Chairman of the Committee of Arrangement and Reception, before reading the list of Delegates, congratulated the Association on the recurrence of its anniversary. Seven years had elapsed since the preliminary meeting of the Convention, which recommended the organization of this National Congress; and, as New Yorkers, the committee indulged in feelings of proud satisfaction at the triumphant success which had attended so important a movement, originating in their State. The labors of the learned body had been most arduous, but the result had not disappointed the expectations of the friends of reform and of progress in their profession. Mighty objects were aimed at; important achievements had already been accomplished; and a guarantee was afforded of the eventual fulfilment of the desires of those whose aspirations for the advancement and perfection of medicine, led them to propose the formation of this association, which, representing the whole fraternity throughout our wide-spread country, assembled annually, for scientific discussion, and to consult upon matters pertaining to the general welfare of the entire faculty. The five published volumes of their Transactions afforded abundant and conclusive evidence of the zeal by which they were actuated, and the ability which characterized the scientific labors of members. The meeting for this year had been generally anticipated with peculiar interest. An unusually large attendance had been expected. Numerous papers would be presented, and valuable reports were to be rendered; much of their time would be required for the consideration of subjects of grave importance. He assured them that their advent had been looked for anxiously, and their colleagues had been desirous to manifest their appreciation of the course in which they were engaged, and their estimation of the favor conferred by the Association in selecting this metropolis as the place for holding the present session. In the name of the united Profession of New York the Committee tendered them a sincere, hearty, and cordial welcome to their City.

Dr. S. then called over the list of Delegates, and announced that a majority were present.

The Vice-Presidents and Ex-Presidents of the Association were requested to take their seats on the platform with the President.

On motion, a recess of fifteen minutes was taken to allow the delegates to select one of their number from each State, as a committee to nominate officers for the ensuing year.

The following Nominating Committee was announced:

| | |
|---|---|
| Michigan, - - - | Dr. Henry Taylor |
| New Hampshire, - - | " Josiah Crosby |
| Maine - - - - | " Isaac Lincoln |
| Connecticut, - - - | " Archibald Welsh |
| New Jersey, - - - | " Lewis Condict |
| Maryland, - - - | " Joel Hopkins |
| Indiana, - - - - | " Joseph Somes |
| Rhode Island, - - - | " Hiram Allen |
| Kentucky, - - - | " J. B. Flint |
| Tennessee, - - - | " J. B. Lindsley |
| Virginia, - - - | " Thos. P. Atkinson |
| New York, - - - | " J. M. Smith |
| Missouri, - - - | " Charles A. Pope |
| Iowa, - - - - | " J. C. Hughes |
| Ohio, - - - - | " R. L. Howard |
| Georgia, - - - - | " Henry F. Campbell |
| South Carolina, - - | " Henry R. Frost |
| Alabama, - - - | " G. A. English |
| North Carolina, - - | " J G. Tull |
| Illinois, - - - | " N. A. Davis |
| Massachusetts. - - | " A. L. Pearson |
| Pennsylvania, - - | " F. West |
| Vermont, - - - | " E. S. Carr |
| Delaware, - - - | " H. F. Asken |
| District of Columbia, - | " Thos. Miller |

Dr. Atlee, of Pennsylvania, moved that immediately after the report of the Nominating Committee, the President be called upon to read his address, but, at the request of Dr. Condie, withdrew his motion until the report of the Treasurer should be read.

Dr Pope, of Missouri, extended an invitation to the Association to hold their next annual meeting, for 1854, at St. Louis.

Dr. Condie, of Pennsylvania, said he was instructed to invite the Association to hold their next annual meeting in Philadelphia.

Dr. Hays, of Pennsylvania, although desiring that the Association should meet in Philadelphia, was forced to give his voice for next year in favor of St. Louis. He hoped that, at their next meeting, the claims of Philadelphia would be remembered.

The motion to meet at St. Louis was put and carried.

Dr. Condie, of Pennsylvania, Chairman of Committee on Publications, submitted a report from the Committee, with resolutions appended, making the assessment for the present year $5; authorizing the Committee to decide upon the terms at which the volume of Transactions for this year shall be furnished; and further authorizing them to take such measures in relation to disposal of the copies as they may deem expedient. Dr. Condie stated that a valuable paper would be submitted at this meeting, the mere illustrations of which would cost $1,000 for printing.

The resolutions were adopted.

The Treasurer's Report showed the total receipts for the past year to be $1,905; paid out, $2,015; balance due to Treasurer $120.

On motion of Dr. Condie, the Committee was authorized to furnish the Chairmen of Committees on Epidemics with extra copies of their reports, respectively, at the expense of the Association.

The Secretary read communications inviting the Association to visit University Medical College, the Anatomical Museum, and Bloomingdale Lunatic Asylum.

Dr. F. C. STEWART presented a report, recommending the admission of Dr. Marshall Hall of London, Surgeon Mower, U. S. A., Surgeons Bache, Pinckney, Brownell and Simpson, U. S. N., Drs. Leonard and Betton, Florida, Hon. Dr. Bartlett, N. Y. Senate, Dr. Harris, Canada, Dr. Rodder, Canada West, Drs. McIlvaine and Pittman, American Medical Society, Paris, to participate in the proceedings by invitation.

On motion of Dr. Cox, a Committee was appointed to wait on Dr. Marshall Hall, and conduct him to a seat on the platform.

### DR. WELLFORD'S ADDRESS.

The President's address was an eloquent exposition of the objects of the Association—a rehearsal of the good it had done, and an indication of certain modes by which it may do still more. The Society has been in existence some seven years. It grew out of a severely felt want of an organization of the scattered medical forces. A Convention assembled in this city, at the suggestion of the New York Medical Society, to take into consideration the condition of the profession, and to adopt some concerted action. Although the call was imperfectly circulated, about one hundred delegates from nineteen States of the Union responded to it by their personal presence. And they resolved to institute a National Association for the protection of their interests, the maintenance of their honor and respectability, the advancement of their knowledge and the extension of their usefulness. In the untried circumstances of their early history imperfections in the plan of organization were inevitable. The President advised careful and gentle revision, but deprecated all desultory innovations or sudden and radical changes.

There are those who affect to believe that the Association has hitherto effected nothing. In answer, the Doctor referred to the fact that it has aroused attention to the imperfections of medical cultivation—that it has demonstrated the necessity of raising the standard of medical acquirements, and done much through its potent leverage to raise the profession to its proper level. It has secured in many Colleges a lengthened course of lectures, and encouraged the well-disposed to require of the candidates for graduation higher attainments in general and professional knowledge. It has stimulated professional ambition, and drawn out from quarters whence no other means would have elicited it, highly important contributions to medical science. It was the action of the Association, in its early days, which directed the attention of the Legislatures of several States to the high importance of collecting aad measuring their vital statistics. Following its suggestion, laws have been passed in New York, Massachusetts, Rhode Island, Connecticut, Michigan and Illinois, we believe, and in several of the Southern States—not always of the most practicable sort or the best that could be devised, yet in every case highly honorable to the intentions of their framers, and likely to lead to more perfect systems—laws which provide for the registration of all the births, deaths and marriages within their several borders. In obedience to this recommendation of the Association, and in consequence of it, the Convention which recently sat in Virginia, for the purpose of amending the State Constitution, made the passage of a registration act obligatory on her Legislature. In compliance with that requisition a law was passed at its late session, the first under her new Constitution.

Another tangible and note-worthy result of the efforts of the Association, is the action of the Fedaral Government with reference to the adulteration of drugs. At the meetings in Baltimore in 1838, its attention was directed to the immense amount of adulterated and sophisticated drugs prepared in foreign countries, and imported into our own. Congress promptly responded to the recommendation of the Association, and in an act, approved June 26th, 1848, Inspectors were appointed, and the passage of any drug or medicine so adulterated or deteriorated as to be inferior in strength and purity to the standard established by the United States, London, Edinburg, French and German Pharmacopœias and Dispensatories, positively prohibited. The Examiner of Drugs in the New York Custom House, within five months after this law went into operation, had condemned and rejected no less than 13,000 lbs .of rhubarb, 2,500 lbs. of opium, 7,200 lbs. of jalap, 1,414 lbs. of gum gamboge, 1,400 lbs. of senna, 30,000 lbs. of spurious yellow bark, 3,000 lbs. of iodine, and 1,700 lbs. of myrrh, all of which, but for this law, would have found its way throughout our extensive country, and to the bedsides of the sick and the suffering, to mock them with their inertness, or poison them with their unsuspected strength. A Senator in Congress well remarked that, if the National Medical Association had advised no other reform than this, its labors would entitle its members to the gratitude of the country. Dr. Edwards, who was employed by the Secratary of the Treasury to visit the principal ports and ascertain the working of the law, found them to be : 1. An elevation in the quality and purity of the medicinal agents imported. 2. An entire prevention of adulterated and deteriorated drugs, &c., from entry and use. 3. No embarrassment to the honest importer and dealer. 4. An increased revenue. 5. Protection to the medical profession and community, and increased confidence in and a desire for the continuance and faithful application of the law. Spurious and adulterated articles now are almost entirely excluded from our markets, with the exception of those of home manufacture. Nor have these increased. Attention having been thoroughly aroused to the whole subject, adulterations cannot be practised with impunity as formerly.

Much has been done, and yet it is only a beginning. The learned

President next addressed himself to the task of indicating the points demanding immediate action.

The city affords advantages which the country practitioner cannot enjoy. In the city, professional men meet daily; every day they talk over affairs pertaining to their profession. Their emulation is kept alive; the ambition of the zealous is never permitted to flag. In the country it is otherwise; a professional consultation is only an occasional event, and anything like daily contact with friendly brethren is out of the question. Yet physicians are but men, and when all the talk of a village is of cattle and rich acres, and of politics, the physician talks of such things, too—grows rusty on medical matters, and becomes *au fait* on politics and farming. In some small degree to remedy this, let local medical societies be planted; and to facilitate their growth, the President advised the appointment of a committee to prepare a form of organization of both local and State societies, and that its adoption be earnestly recommended.

He called attention to the very unsatisfactory state of things which allows a diploma to be considered equivalent to a license to practice, and, indeed, tolerates anybody as a practitioner who chooses to dub himself a doctor—favored the invocation of State legislation on the subject, and the establishment of Licensing Boards to be entirely distinct from all bodies that grant diplomas.

He invited a recommendation to the authorities, charged with the duty of such action, that will shield the public from the home production of adulterated medicines. All that could be done to prevent the importation of these nuisances, the Federal Government has done; but there remain necessary such enactments by the several States as will make disreputable and unprofitable the business of preparing for the market inert medicines or drugs of a strength inferior to the officinal standard.

Dr. John A. Lamb, of Pennsylvania, is the only one who has been appointed under power of the resolution adopted in 1852, to travel in Europe, and report on foreign medical affairs. With a touching tribute to the memory of Drs. Drake and Horner, members who have died during the twelvemonth, and a word of advice fitly spoken, the President concluded his Address.

Dr. Hays, of Pennsylvania, moved the thanks of the meeting be presented to the President for his elegant, appropriate and eloquent address, and requested a copy for publication in the Transactions of the Association. Carried.

The Secretary read a resolution passed by the Medical Society of Virginia, recommending the appointment of a well-qualified chemist to analyze the most prominent nostrums of the day, and publish the results monthly in the leading newspapers of each State. Also, a communication from the President of the American Medical Society at Paris, appointing Drs. Pittman, Walton and McIlvaine to attend this meeting.

On motion of Dr. Atlee, the Committee on Publication were directed to send a full set of the Transactions of the Association to the Medical Society in Paris.

A communication was received from Dr. RAMSAY, of Georgia, inclosing documents on personal matter, which were laid on the table.

The Committee on Nominations reported the following officers for the ensuing year.

For *President*—Dr. JONATHAN KNIGHT, of Connecticut.

*Vice Presidents*—Drs. USHER PARSONS, of R. I., LEWIS CONDICT, of N. J., HENRY R. FROST, of S. C., R. L. HOWARD, of Ohio.

*Secretaries*—Drs. EDWARD L. BEADLE, of N. Y., and EDWIN L. LEMOINE, of Missouri.

*Treasurer*—Dr. D. FRANCIS CONDIE, of Pennsylvania.

The Committee reported St. Louis, Mo., as the place to hold the next annual meeting.

The report was adopted.

Drs. Gooch, Watson and Atlee were appointed a Committee to conduct the President-elect and other officers to their seats.

Dr. KNIGHT, on taking the Chair, returned thanks for the honor conferred on him.

Dr. ATLEE, of Pennsylvania, moved a vote of thanks to the late President, Dr. Wellford, for his dignified, courteous and efficient manner in the Chair. Carried unanimously, the members rising.

A vote of thanks was also passed to the retiring Secretary, Dr. Gooch.

On motion of Dr. HOPKINS, of Maryland, it was resolved that no member speak more than ten minutes on any subject at one time.

Dr. STEWART, on part of Committee of Arrangements, suggested that the Association meet each day from 9 to 12 A. M., and from 1 to 4 P. M.

On motion of Dr. GOOCH, the meeting adjourned to 9 A. M. on Wednesday.

*Second Day, May 4th.*

The Association met at 9 A. M. There was a large attendance of delegates. President in the Chair.

Dr. E. L. BEADLE, Secretary, read the minutes, which were adopted.

Dr. COX, of Maryland, moved for a re-consideration of the vote adopting the minutes, to allow of a correction in the Special Report of Committee of Arrangements, inviting Drs. Pinckney and Bache, U. S. N., to take part in the proceedings. These gentlemen had a right to sit as delegates from the Army and Navy.

Dr. STEWART explained, and read from the 2d article of the Constitution relating to delegates.

Dr. WATSON, of New York, stated they had always been received as delegates.

The motion to reconsider was put, and lost.

Dr. COX hoped the Association would not take any action that would give offence to the Army and Navy Medical Institutions. He moved that Drs. Pinckney and Bache be received as regular delegates.

Dr. PINCKNEY, U. S. N., asked to be heard, and claimed his right to a place as delegate, having been formerly received as such, and signed the constitution.

Dr. F. C. STEWART offered to following as an amendment to Dr. Cox's motion :

*Resolved,* As the sense of this Association, that under its present Constitution delegates can be received from the U. S. Army and Navy Medical Bureaus, when appointed by the Chief of the Army and Navy Medical Bureaux.

The amendment was adopted.

Dr. STEWART, from the Committee of Arrangement, reported that several members had registered their names since the report of yesterday.

Dr. COX, of Maryland, proposed Dr. Borland, of Arkansas, as a member by invitation.

Dr. SPENCER proposed Dr. Hurd, of New York, but, on explanation from the Chair, withdrew the proposition.

Dr. C. D. MEIGS, of Philadelphia, presented a report on "Acute and Chronic Diseases of the Neck of the Uterus," with a request that it should be referred to Committee on publication without reading.

The report was adopted and referred.

Dr. COVENTRY, of New York, moved for a suspension of the order of business, to take up the subject of proposed amendments to the Constitution.

Dr. CASH, of New York, moved that the resolution be laid on the table, and the regular order of business be proceeded with. Carried.

The Secretary proceeded to call up reports from special committees. Out of a list of twenty-seven committees only four reported. The rest either did not answer, or were not ready.

Dr. CONDIE, of Pennsylvania, Chairman of Committee on Causes of Tubercular Diseases, stated that in consequence of his duties as Treasurer and Chairman of Committee on Publication, he had not been able to complete his report. On motion of Dr. ATLEE, the Committee was continued.

On the part of Dr. Porcher, of South Carolina, Dr. CONDIE stated that he had sent to him the report of Committee on "Toxicological and Medicinal Properties of our Cryptogamic Plants," but with the request that it would be left, for further additions, to the Committee. The Committee was continued.

Dr. G. EMERSON, of Pennsylvania, Chairman of Committee on "Agency of the Refrigeration produced through upward radiation of heat, as an exciting cause of Disease," presented his report, which was referred to Committee on Publication, and read an abstract. The sanitary lesson designed to be inculcated in this paper, is the importance of guarding against exposure to the refrigerating effects of nocturnal radiation, especially in sickly places and during epidemic periods. The means of effecting this are shown to be extremely simple and always at hand, as anything will answer the purpose which may be interposed to cut off the view of the open sky, and thus prevent "upward radiation."

Dr. H. F. CAMPBELL, of Georgia, presented a report on Typhoid Fever, which was referred to Committee on Publication. Dr. Campbell said that he had little experience in the actual treatment of typhoid fever, as it rarely prevails in the district where I am lecated. I have therefore given a condensed history of the existing pathology regarding it, set forth by other writers, accompanied with my own opinion that the disease lies and has its origin in the ganglionic system of nerves. If you divide some of the superior branches of these nerves, there is an immediate ecchymosis of the eye different from the ganglionic congestion observable during typhus fever. I have called attention to the existence and causes of the maculated spots which appear upon the surface in the one variety of fever and extend through the alimentary canal in the other; and reason that the latter morbid appearances are the result of the diseased ganglionic plexus extending from the superior cervical vertebræ through the vertebral column to the ganglions of the sacrum. In referring typhoid fever to this cause, I have recorded the appearances presented in the pharyngeal plexus, the larynx, œsophagus, stomach and duodenum, (which is separated in a great degree from the influence of the cerebro-spinal system,) and I have then pointed out the existence of the ulcerations of the lower portion of the ilium, and to a great extent, as a reason for my belief. I have traced the different appearances observed in typhus fever. I have examined the theory of Woods upon the deficiency of fibrin in the blood, and endeavored to show that typhus and typhoid fevers are quite distinct diseases.

Dr. Atlee, of Pennsylvania, Chairman of Committee on Epidemics in New Jersey, Pennsylvania, Delaware and Maryland, said that the Committee were unable to report, in consequence of not receiving any communications from gentlemen in those States. The Committee had only received two communications—one from Philadelphia and one from Franklin county, Pennsylvania. He called upon gentlemen practising in these States to furnish the Committee with the results of their experience.

Prof. Chalmer, Chicago, moved the following:

*Resolved*, That this Association earnestly recommend to the local Societies in different portions of our country, to appoint Committees, whose duties it shall be to record the prevalence of epidemic or other diseases, and the general state of health in their respective localities, and to transmit said reports to the Committees of the Society on epidemics, through the State Societies where they exist.

*Resolved*, That the Secretaries be requested to secure a wide publicity to the above resolutions, by such means as they may deem proper.

The resolutions were adopted.

Dr. Wellford announced that Dr. Haxall, of Virginia, Chairman of Committee on Epidemics in Virginia and North Carolina, and Dr. Bolling, of Alabama, on Epidemics in South Carolina, Georgia, Florida and Alabama, had resigned their appointments as Chairmen of such Committees.

Dr. Sutton, of Kentucky, presented a report on Epidemics in Tennessee and Kentucky, which was referred to Committee on Publication.

Dr. Pitcher, of Michigan, presented a report on the subject of Medical Education, which he was requested to read at length. The report was a long and able document, containing many valuable suggestions to prevent the spread of Quackery, and on the best means of training the medical student. The Committee proposed that all candidates for degrees shall have studied at least three years, and recommend the extension of lecture seasons to six months. The Committee repeated their high opinion of the benefits to be derived by students from bedside experience, as superior to lectures and flitting hospital visits, and suggested a supplementary school of practice. They would not discourage medical schools through the country, but foster them as useful, and trust to the private instructor and the hospitals as schools of practice. The Committee asked leave to conclude their report by presenting the following resolutions:

*Resolved*, That the Association reaffirm its formerly expressed opinions, on the value and importance of general education to the student and practitioner of medicine, and that it would gladly enlarge its rule on this subject, so as to include the Humanities of the schools, and the Natural Sciences.

*Resolved*, That in the opinion of this Association, a familiar knowledge with the elements of Medical Science should precede Clinical instruction.

*Resolved*, That in order to accomplish the latter, the Hospitals, when elevated to the rank of Schools of Practice, and the intelligent private preceptor, are the most efficient instrumentalities to be used for that purpose.

On motion of Dr. ATLEE, the report and resolutions were adopted.

The Committee on Volunteer Communications reported, through Dr. Joseph M. Jmith, of New York, upon the number of contributions received. Dr. Smith said that the Committee had awarded one prize of $100 to Dr. Waldo J. Burnett, of Boston, Mass., for his treatise upon "The Cell; its Physiology, Pathology, and Philosophy"—"*Natura in minimis maxima est.*"

Another prize of $100 had been awarded to Dr. Washington L. Atlee, of Philadelphia, for his treatise upon "The Surgical Treatment of Fibrous Tumours of the Uterus." "*Palmam qui meruit ferat.*"

Dr. ALDEN MARCH, of New York, made a verbal abstract of his paper on "Diseases of the Hip Joint," which was favorably reported on by the Committee; and, on motion of Dr. SMITH, he was requested to read the paper, during recess to-day, in Crosby street Medical College.

Dr. PALMER, of Chicago, moved the following:

*Resolved*, That this Association earnestly recommend to the local Societies in different portions of our country, to appoint Committees, whose duties it shall be to record the prevalence of epidemics or other diseases, and the general state of health in their respective localities, and to transmit said reports to the Committees of the Society on Epidemics, through the State Societies where they exist.

*Resolved*, That the Secretaries be requested to secure a wide publicity to the above resolutions, by such means as they may deem proper.

The resolutions were adopted.

Dr. ATLEE, Pa., called the attention of the Association to resolutions passed at the last annual meeting of the Association, for purchasing a suitable stone for the Washington Monument.

Dr. Blatchford, N. Y., offered the following:

*Resolved*, That the suggestions in the President's Address, touching the licensing power, be referred to a Committee of five, of which Dr. Wellford shall be the Chairman, to prepare some plan whereby the subject may be brought fairly before the Profession, and, if deemed advisable, that the Legislatures of the several States may be memorialized to carry out the recommendations of this Association—the Committee to report at the next meeting of the Association.

Dr. Garnett, Washington, D. C., proposed the following as an amendment:

*Resolved*, That a Committee of five be appointed by the President of this Association to prepare a memorial to the Legislative bodies of the several States of the Union, praying that a law be passed prohibiting the Faculty of any medical institution which may at present exist, or which may hereafter be established within the limits of said State, from conferring the degree of Doctor of Medicine upon any candidate for graduation who has not previously graduated at some literary institution, or who, upon examination by a competent Board, is not found to possess a good English and Classical education.

*Resolved*, That regarding the present term of preparatory study adopted by the Medical Colleges of the United States, too limited to enable students of medicine to acquire such a competent knowledge of the profession as should entitle them to receive the degree of Doctor of Medicine, a recommendation be incorporated in said memorial that some Legislative action be also had upon this subject.

*Resolved*, That it be the duty of the President and Secretary of this Association, to transmit, through the Executive heads of each State, a copy of said memorial to their respective Legislative Assemblies, and that a Circular be addressed to the members of the Medical Profession, resident at, or convenient to the seats of Government of the several States, requesting them to use every practicable exertion consistent with the honor, dignity, and good repute of the profession, to procure the passage of a law in accordance with the foregoing resolutions.

On motion of Dr. Cash, the resolutions and amendments were laid on the table, to be taken up at an early period, and the Association took a recess for one hour.

*Afternoon Session.*

The Association met at 2 o'clock, P. M. The Secretary read an invitation, from Dr. Griscom, to visit the New York Hospital.

Dr. Stewart, on the part of the Committee of Arrangements, moved that Dr. Robert R. Halley, of Beyroot, Syria; Dr. James G. Cooper, Washington Territory, and Dr. H. Williams, from Southern Illinois, be invited to take part in the proceedings. Carried.

The following resolutions, offered by Dr. Charles Lee, a permanent member, were read, in his absence, by the Secretary.

*Resolved*, As the sense of this Association, that those Medical Colleges which give two courses of lectures annually, each of which counts as a separate course, have virtually violated and forfeited their Charters, which do not contemplate but one annual session, (thus making two Colleges out of one.)

*Resolved,* That the practice in question is calculated to lower the standard of attainment in the profession, and subjects those who countenance it to the imputation of acting from mercenary motives.

*Resolved,* That no delegates shall hereafter be received by this Association, from any Medical Schools which give two courses of lectures annually; each of which counts towards a degree.

On motion of Dr. ATLEE, of Pennsylvania, the resolutions were laid on the table.

Dr. STEWART read the following resolutions, offered by Dr. Stephen W. Williams, of Mass., a permanent member:

*Resolved,* That the thanks of this Association be presented to Dr. WINSLOW LEWIS, of Boston, a member of the Massachusetts Legislature, for the bill which he has presented and endeavored to sustain, providing that "no druggist, apothecary, or person engaged in manufacturing medicines, or compounds to be administered as medicines, (except such as are published in standard works of chemistry, materia medica, or pharmacopœia,) shall offer the same for sale in any way, till he has filed a complete recipe in English, sworn to before a legal authority constituted for such purpose."

2. *Voted,* That a Committee be appointed by this Association for the purpose of petitioning Congress and State Legislatures to enact regulations and laws similar to the above.

Also, as we are constantly called upon to deplore the ravages of death among the illustrious and worthy members of our profession throughout the United States:

*Resolved,* That a Standing Committee be appointed by this Association to procure memorials of the eminent and worthy dead among the distinguished physicians of our country, both in and without the pale of the Association, and present them to this Association for publication in their transactions.

The first resolution, relating to Dr. Lewis, was adopted.

To the second Dr. COX, Md., moved an amendment, confining the memoirs to distinguished members of the Association.

Dr. MORGAN, Washington, thought the Association would not be able to meet the increased expense such an addition to their transactions would cause. He moved to lay on the table. Carried.

Dr. BUCK, N. Y., read a paper on morbid growths within the larynx, and exhibited a specimen, with report of the case. Referred to Committee on Publications.

Dr. J. K. MITCHELL, Pa., offered the following:

*Whereas,* The claim of Naval Medical officers to a defined rank, assimilated with the grades of officers of the line of the Navy, has not yet been decided by Congress, therefore

*Resolved,* That the President of this Association appoint a Committee of three, which is hereby instructed to communicate to Congress, through the presiding officer of each House, at the commencement of its next session, an expression of the interest felt by the American Medical

Association of the United States, for their professional brethren employed in the Navy, as set forth in resolutions unanimously adopted at several previous sessions of this body.

Dr. JACKSON, of Philadelphia, seconded the resolution.

The President stated that the bill had passed one branch of our National Legislature.

Dr. PINCKNEY, U. S. N., made a statement of the action had in the National Legislature.

The resolution was put and adopted.

Dr. STEVENS, of New York, moved that the President be Chairman of the Committee. Carried.

Dr. HOOKER, of Connecticut, offered the following, which was adopted :

*Resolved,* That the delegates from the several States be requested to appoint a Committee, who shall aid the Committee of Publication in procuring subscriptions, and in distributing the annual transactions of the Association.

Dr. BOLTON, Virginia, moved a resolution expressing the warm commendation of the Association towards the medical department of Michigan University, for its co-operation in elevating the standard of the profession.

A discussion ensued, in which Drs. PALMER and BOLTON advocated the resolution, and Dr. HOOKER and others took ground against it. Finally, the resolution was withdrawn.

Dr. Muran presented the following report, in relation to the necessity of each emigrant ship being provided with a surgeon :—

The undersigned, Chairman of a Committee appointed by the American Medical Association, to memorialize Congress upon the importance of enacting, as an act of duty and humanity, a law making it obligatory upon all vessels conveying emigrants to have a regular surgeon on board, respectfully reports : That the memorial signed by all the members of the Committee, the President and Secretaries of the Association, was, through the medium of the Hon. A. P. Butler, Senator from South Carolina, presented in May, 1852, and was referred to the Committee on Commerce. It is with much regret that the undersigned has to report that the Committee on Commerce, having, it is presumed, other subjects of a much graver import in a pecuniary point of view, but surely not in a humane and philanthropic one, made no report. It was not contemplated that any action would have been taken on the subject at the late period of the session of 1851 ; but sanguine hopes were entertained that some report would have been made in the session of 1852. The undersigned, in common with every good citizen, has a respectful consideration for our legislators and public functionaries, but must honestly confess that the legislation growing out of outward pressure, for ulterior purposes of individual or political aggrandizement, renders it difficult to have such prompt and efficient action as should be reasonably expected upon questions involving sacrifice, advancement, or Hygienic improvement. The undersigned, however, is fully impressed that no intentional disrespect was meant by the Committee on Commerce in allowing the memorial to remain in their archives unnoticed; and now that the Presidential election, and other collateral subjects, incidental or having a bearing thereto, are settled, it is but reasonable to suppose that the Senate and House of Representatives would consider any measure as regards science or medical association; and likewise, if possible in legislation, adopt the same. There has been every assurance given to the undersigned, that the measure will have a proper consideration at the next session of Congresss, and therefore he asks that the Committee be continued, and considered as reporting progress.

THOS. Y. SIMONS, M. D., Chairman.

The foregoing report was accepted, and the Committee directed to prepare a memorial to Congress.

The following report was then presented:

The undersigned, Chairman of a Committee of the American Medical Association to memorialize Congress in accordance with a resolution of Dr. Fulton, of Georgetown, Kentucky, to have the medical statistics of the United States census printed separately, for the use of the medical profession, respectfully reports:—

That a memorial was drawn up, and signed by the Committee and the President and Secretary of the American Medical Association, and was placed in the charge of the Hon. Dr. Jones, a member of the Association, and of Congress, to be presented to the House of Representatives. From information received from him, it seems, among other objections, a grave one was offered, viz:—the want of scientific arrangements, and the unreliability of the returns in general. In other words, that they were of such a character as to add little to the usefulness of the profession or the honor of the country. The undersigned, respectfully, on the present occasion, calls the attention of the members of the Association to the great importance of using their best influence to induce the legislatures of their respective States to establish a registration of births, marriages and deaths; a measure of incalculable value as regards vital statistics. The experience and gradual progressive improvement in the reports of Massachusetts, clearly demonstrate that while at first they were comparatively imperfect, yet much information was obtained, and every year the reports have become more satisfactory.

THOS. Y. SIMMONS, M. D., Chairman.

The report was adopted, and the Committee continued.

Dr. PEASLEE, of N. H., offered a resolution, setting forth that various systems of irregular practice were encouraged by the regular medical Colleges, in giving diplomas to such as subsequently engaged in them, and moving that the Colleges refuse to admit all persons who intend to engage in any other than the regular practice of medicine, to an examination.

Dr. SAYRE, of N. Y., considered the resolution would not meet the intention. He moved an amendment, petitioning Congress for the passage of an act, empowering the Faculty in any College, to publicly withdraw diplomas from graduates who had, in their judgment, forfeited a right to the same.

A warm discussion ensued, in which Drs. HOOKER, Conn., ATLEE, Pa., PEASLEE, and others, took part.

On motion of Dr. GOOCH, the resolution and amendment were laid on the table, and the Association adjourned to Thursday morning at 9 o'clock.

*Third Day, May 5th,* 1853.

The Association met at 9 A. M., Dr. JONATHAN KNIGHT, President, in the Chair.

The Secretary, Dr. E. L. BEADLE, read the minutes, which were approved.

Dr. STEWART, Chairman of Committee on Arrangements, reported the names of several delegates who had registered since last report.

Dr. W. HOOKER, Connecticut, offered the following, which was adopted:

*Resolved,* That a Committee of Five be appointed, whose duty it shall be, in compliance with the suggestions of our late President, Dr. Wellford, to report our plans of organization for State and County Societies, and that the Committee be requested to report, if possible, during the present meeting of the Association.

Dr. ZEIGLER, Pennsylvania, offered the following, which was referred to Committee on Organizing State and County Societies:

Inasmuch as the universal aggregation and organization of the members of the medical profession in the United States has long been a desideratum but partially attainable until the adoption of the present mode of organizing local and general societies in imitation of and in conformity with the confederacy of this country; and whereas, notwithstanding this consolidation has thus been more completely and perfectly effected, a very large portion of the profession still remain isolated and unassociated, thus retarding and preventing the more rapid attainment of those exalted objects involved in this universal professional union in one great brotherhood; and inasmuch as the present general mode of perfecting further and final professional aggregation and consolidation is comparatively imperfect, inefficient, and protracting, and hence will require an almost indefinite period for its full accomplishment and final consummation and completion; and inasmuch as some more general and systematic effort for the speedy and positive realization of this highly important object is yet greatly desirable; therefore

*Resolved,* That the American Medical Association hereby reiterate the repeatedly previously expressed desire for the immediate formation and organization of County and State Medical Societies in every part of the country in which they have not yet been established.

*Resolved,* That every County Medical Society be and is hereby recommended to dispense with the present system of acquiring members by the previous pro-formal manifestation of personal desire on the part of applicants for such association, and to substitute therefor that of the immediate and voluntary election to membership therein, of every unassociated eligible physician.

*Resolved,* That all physicians thus voluntarily elected, and subsequently neglecting or declining to respond to and unite themselves with the general profession, shall be considered as estimating their own personal views, and private relations and interests, above and in opposition to those of the profession generally, and, as thus antagonistic to its exalted objects, cannot therefore consistently expect the continued enjoyment of the usual rights and privileges of professional intercourse and fellowship.

The Secretary read a notice, requesting delegates from any States which were not represented on the Committee on Nomination, to elect representatives to sit with the Committee at 56 Bleecker street.

Dr. N. S. DAVIS, of Illinois, reported at length, on the Medical Literature of 1853. There are now published in the United States twenty-eight medical periodicals, of which four are issued quarterly, six bi-monthly, fifteen monthly, two semi-monthly, and one weekly. One of the monthlies is published in the German at New York, and one in French, at New Orleans. Of the aggregate number of pages published, about one-half were original matter. This aggregate consists of the record of cases occurring under the observation of their writers, of which a very large proportion lose their value for lack of that fullness of detail and scope, which are essential to make them reliable data for the abstraction of practical deductions—articles embodying the statistical results of certain diseases and surgical operations, and essays on special subjects, and the details of experimental inquiries—of all which classes some of the more important specimens were named in the report. It is believed that a decided improvement has taken place in this department of medical journalism during the past year. It is shown most distinctly in the more frequent reports of the use of the microscope and its application to physiological and pathological researches. But the report scores the journals for an abundance of material furnished of another order—crude, ill-digested essays, illogical, incomplete and consequently mischievous articles, which serve to advertise the writers' names and residence, and, unwittingly, their ignorance.

The Review department of our medical periodicals is of all the most defective. There are a few honorable exceptions, but the large majority afford little more than a few meagre pages of booksellers' notices, serving to advertise the work whose title is given, and seldom affording any impression whatever of the character and contents of the book named.

The number of journals in this country is greater in proportion to the population than in any other. Whether we are gainers in their quality from this fact is very questionable, but this, at least, it secures —a greater number of professional readers. A very copious contribution to our medical literature is made in the form of the transactions of the State, County and other Medical Societies. The report hints that their character is decidedly superior to that of the average of the original papers contributed to the journals.

Among the more valuable monographs that have issued from American authors during the year, are, the treatises of Dr. Swett on the Chest; Dr. Flint on Continued Fever; Dr. Edward Coale, on Uterine Displacements; Dr. H. H. Smith's Operative Surgery; Dr. Piper's Illustrated Surgery; Dr. Horace Green's Polypi of the Larynx and

Œdema of the Glottis; Dr. Biddle's and Dr. Tully's Materia Medica; Dr. Dickson's Life, Sleep and Death; and Dr. Mackall's Notes on Carpenter's Physiology. Besides these, many new editions of standard American works, and the revision and translation of many of foreign origin, show that there is no lack of patronage of our home literature, nor a lack of readers for professional works, either domestic or foreign. In matters of minutiæ, in the details of analysis and of nice scientific discrimination, we have looked abroad for our medical teachings. But in the details of practice, in bold, independent, inventive and energetic thinking on medical topics, we are not copyists, nor in any respect imitators.

The defects of our medical literature are very obvious and readily traceable to their causes—to wit, a lack on the part of medical writers of sufficient preliminary education, the absence of clear and definite perceptions of the fundamental principles of physiology and pathology, defective modes of investigation, and the epidemic haste shared by them with all other writers in our country, with which their productions are surrendered to the press. These difficulties will be remedied when the public sentiment of physicians has become what it ought to be; when medical critics are honest in expressing their convictions, and medical men avail themselves of the benefits that must accrue from the organization of their forces into local, State and National Associations.

The following he indicated as the principal means of advancing the standard of Medical Literature: 1. To adopt such means as would be calculated to increase preliminary study on the part of students. 2. Greater strictness on the part of Medical Colleges in their examinations. 3. Offering premiums for the best original Medical Essays by State and County Medical Societies. 4. Formation of Medical Libraries. 5. To labor for an International Copyright. The future is bright for us, the past full of eloquent lessons. If we heed them and do for the future what we ought, American Medical Literature will yet attain to an elevation corresponding with our country's social and political destiny.

The Secretary read a communication from Robert Kelly, Esq., inviting the Association to visit the House of Refuge for reformation of juvenile delinquents.

Dr. S. JACKSON, of Pennsylvania, stated that he had received a paper from one gentleman on Electricity, as applied to predictions of meteorological changes.

Dr. MCNAUGHTEN moved to refer to Committee on Spirit Rappers. Laid on the table.

Dr. YANDELL, of Kentucky, offered the following, which was adopted:

*Whereas*, By the dispensation of an inscrutable Providence, Dr. DANIEL DRAKE has been removed since the last meeting of this Association from the scene of his earthly labors,

*Resolved*, That in the death of Dr. Drake the American Medical Association has lost one of its most honored members, and the American medical profession one of its brightest ornaments.

*Resolved*, That his steady devotion to his profession, through a long life, his zeal, activity and unceasing efforts to advance its interests, afford an example worthy of the imitation of every young physician.

*Resolved*, That this Association will cherish the memory of Dr. Drake for his many virtues, and for his labors which have adorned and elevated our profession.

The resolutions were adopted by a rising vote.

The President read a resolution, offered by Dr. CLEVELAND, of Vermont, calling for the appointment of a Committee to investigate the value of Galvanism as a therapeutic agent.

On motion of Dr. GOOCH, the resolution was referred to Committee on Nominations.

Dr. WELLFORD, of Virginia, stated that the State of Louisiana was not represented, and moved that Dr. Douglass, of Louisiana, who was present, be invited to take his seat as a delegate from that State. Carried.

On motion of Dr. PALMER, of Virginia, the resolution offered yesterday, from the Virginia Medical Society, recommending the appointment of a Chemist, and laid on the table, was taken up for consideration.

Dr. PARKER, of Virginia, offered the following as an amendment:

*Resolved*, That this Association recommend Congress to consider the propriety of passing a law compelling all importers of nostrums to state upon all compounds thus imported their true constituents, and in English.

*Resolved*, That the Secretary be instructed to forward a copy of these resolutions to the Executive of the general Government.

An amendment to strike out all after the word "English," was accepted.

The first resolution was read by the Secretary.

Dr. BOND, of Baltimore, objected to the resolution, because it could do no good, and was likely to do a great deal of injury. He moved to lay on the table, but withdrew it to allow discussion.

Dr. HOOKER, of Connecticut, said the facts from the State of Maine would answer the objections of Dr. Bond. An act had been passed there which was found to be a death-blow to this system of quackery. But on petition of the people and clergy, who were in favor of encouraging it, the law was repealed. He wished parties to know what they swallowed.

Dr. SAYRE, of New York, said that every notice that was taken of any such quackeries only tended to advertise them, and could accomplish no useful purpose. He proposed to lay on the table. Lost.

Dr. COX, of Maryland, never knew any good to follow prohibitory measures against quackery. In the State of Maryland, a set of quacks known as Thomsonians, flourished under prohibitory legislation, and died away when given every freedom and latitude. He believed the surest death-blow to quackery was to treat it with contempt and silence. It was unworthy of any grave notice from this Association. He believed the true remedy against quackery was to be found in attention to their Association.

Dr. BOLTON, of Virginia, drew the attention of the Association to a resolution passed yesterday, thanking Dr. Winslow Lewis for his efforts in supporting a bill before the Massachusetts Legislature. There was a mistake in thinking that the resolution proposed to prohibit the sale of these quack medicines; it only proposed that the ingredients of which they were composed should be set forth on the label. This would divest the medicines of the great charm of mystery which gave them such importance in the minds of the public. They would see that for a few pence they could procure some simple medicine, which was the chief ingredient in the expensive bottle.

Dr. RICHARDS, of Ohio, and Dr. JACKSON, of Massachusetts, followed in opposition to the resolution. The analyzing of these nostrums, by order of the State, would be giving a sanction to their sale.

The reading of the resolution was called for, and a vote taken.

PRESIDENT.—The Nays are the loudest; whether they are the more numerous, I cannot say.

The House was counted, and the resolution laid on the table.

Dr. CONDIE, of Pennsylvania, and Dr. COX, of Maryland, offered the following resolutions, which were adopted by rising.

*Resolved*, That we have heard with sincere regret of the death of our late fellow member, Dr. ISAAC PARRISH, of Philadelphia, who was distinguished by his early and earnest advocacy of the establishment of this Association, by his ardent interest in its proceedings, and by his valuable contributions to its published proceedings.

*Resolved*, That in the demise of Dr. WILLIAM E. HORNER, which has occurred since the last annual session of this body, the American Medical Association has lost one of its illustrious and useful members; and the science of medicine, an indefatigable student and most distinguished teacher.

*Resolved*, That the memory of the gifted subjects of the resolutions, dear as it must ever be to the lovers of medical science universally, will be especialy cherished by this Association, to whose great objects and aims, their last efforts were, during life, promptly and liberally bestowed.

Dr. CONDIE, of Pennsylvania, replied to inquiries received, in reference to back numbers of the Transactions.

Dr. YANDELL, of Kentucky, presented a report received from Dr. S. D. Gross, Kentucky, on the results of Surgical operations for the relief of malignant diseases, which was referred to the Committee on publication. Dr. G. read a brief abstract.

From the facts and statements which have now been presented, embracing the opinions of many of the most intelligent, experienced and distinguished practitioners in different ages, and in different parts of the world, the following conclusions may be legitimately deduced:

*First*—That cancerous affections, particularly those of the mammary gland, have always, with a few rare exceptions, been regarded by practitioners as incurable by the knife and escharotics. This opinion, commencing with Hippocrates, the father of medicine, has prevailed from the earliest records of the profession, to the present moment. Nature never cures a disease of the kind; nor can this be effected by any medicine, or internal remedies, known to the profession.

*Secondly*—That excision, however early and thoroughly executed, is nearly always, in genuine cancer, followed by relapse, at a period varying from a few weeks to several months, from the time of the operation.

*Thirdly*—That nearly all practitioners, from the time of Hippocrates to the present day, have been, and are still averse to any operation for the removal of cancerous tumors, after the establishment of ulceration, rapid growth, firm adhesion, organic change in the skin, lymphatic invasion, the cancerous dyscracy, or serious constitutional derangements; on the ground that, if had recourse to, under these circumstances, the malady almost inevitably recurs in a very short time, and frequently destroys the patient more rapidly than when it is permitted to pursue its own course.

*Fourthly*—That in all cases of *acute* carcinoma, or, in other words, in all cases of this disease, attended with very rapid development and great bulk of the tumor, extirpation is improper and unjustifiable, inasmuch as it will only tend to expedite the fatal result, which, under such circumstances, always take place in a very short time.

*Fifthly*—That all operations performed for the removal of encephaloid cancer and its different varieties, are more certainly followed by rapid relapse than operations performed upon scirrhus or hard cancer.

*Sixthly*—That in nearly all the operations for cancerous diseases, hitherto reported, the history has been imperfectly presented, being deficient in the details which are necessary to a complete and thorough understanding of the subject in each case. This remark is particularly true in reference to the diagnosis of the malady, the minute examination of the morbid structure, and the history of the case after the operation, as to the period of relapse, the time and nature of the patient's death, and the result of the post-mortem examination.

*Seventhly*—That cancerous affections of the lip and skin, now usually described under the name of cancroid diseases, are less liable to relapse after extirpation than genuine cancerous maladies, or those which are characterized by the existence of the true cancer-cell and cancer-juice.

*Eighthly*—That, although practitioners have always been aware, from the earliest professional records, of the great liability of cancer to relapse after extirpation, a great majority of them have always been, and still are, in favor of operation in the early stage of the disease, especially in scirrhus, before the tumor has made much progress, or before there is any disease of the lymphatic ganglions, or evidence of the cancerous cachexy.

*Ninthly*—That many cases of tumors, especially tumors of the breast and testicle, supposed to be cancerous, are in reality not cancerous, but of a benign character, and consequently, readily curable by ablation, whether effected by the knife or by escharotics. It is to this circumstance that we must ascribe the astonishing success which is said to have attended the practice of Hill, of Scotland, Nooth, of England, and Flajani, of Italy.

*Tenthly*—That all operators insist upon the most thorough excision possible; removing, not merely the diseased mass, but also a portion of the surrounding and apparently healthy tissues, as well as all enlarged and indurated ganglions.

*Eleventhly*—That the practice has always prevailed, and still obtains, to save, if possible, a sufficient amount of healthy integument to cover the wound, and to unite, if possible, the wound by the first intention; on the ground that these precautions will tend much to retard, if not to prevent, a recurrence of the disease.

*Twelfthly*—That much stress is laid by writers upon a properly regulated diet, and attention to the bowels and secretions after operation, as means of retarding and preventing relapse.

*Thirteenthly*—That there is no remedy, medicine or method of treatment which has the power, so far as we are enabled to judge of its virtues, of preventing the reproduction of the morbid action after operation, no matter how early or how thoroughly it may be performed.

*Fourteenthly*—That life has occasionally been prolonged and even saved by operation after relapse, as in some of the remarkable cases mentioned in a previous part of this report; but that, as a general rule, such a procedure is as incompetent to effect a permanent cure as a first extirpation.

Dr. BRYAN, of Pennsylvania, rose to correct a statement made yesterday, by Prof. Jackson, that a young gentleman had been allowed to graduate in Philadelphia Medical College, after two weeks study.

Prof. JACKSON stated that he had received the information from a gentleman, whom he named, and had used it as an illustration of his argument.

Dr. GOOCH called up the subject of the graduating pledge, proposed by Dr. Peaslee, of New Hampshire, last evening, and laid over, and proposed the following resolutions:

*Resolved*, That this Association earnestly recommends to all the respectable Medical Colleges of the United States to administer to their graduates, previous to their receiving the diploma, some pledge that they will maintain, to the best of their abilities, the honor and dignity of the profession; and that they will forfeit their degrees, whenever they desert the Orthodox system of medicine.

*Resolved*, That the schools be urged not to graduate any man without requiring him to read the National Code of Ethics and publicly give his consent to abide by it, and that they will reserve to themselves the right to withdraw the diploma, publicly, whenever *the graduating* pledge has been violated.

There were, said Dr. G., two schools which had already adopted such rules. Unfortunately, as things stood, gentlemen were allowed to graduate on payment of their fees, without knowing there was such a thing as a "Code of Ethics" in existence, and permitted to go forth among medical men as their equals in practice. The consequence was, systems of quackery, and want of proper *esprit du corps* among members of the profession, in many instances.

Dr. GARNETT, of Washington, spoke to the resolution.

Dr. ATKINSON, of Virginia, inquired how the diploma could be withdrawn.

Dr. GOOCH replied that the diploma could be always withdrawn, if given under these conditions.

Dr. JOHN H. PHILLIPS, of New Jersey, offered the following amendment:

*Resolved,* That it is the duty of all Boards of Examiners, to whom candidates may apply for examination or approval, to admit none but those who give satisfactory evidence of a good preliminary education, and that a regular Course of Medical Practice will afterwards be pursued, and who shall subscribe to the Code of Ethics adopted by this Association.

Dr. COX, of Maryland, thought the resolution contemplated an extraordinary act of legislation, and there would be great difficulty in applying the principle. The power of revoking a diploma, once given under the legal sanction of a charter, was a dangerous one to be intrusted to any set of men.

Dr. ATKINSON, of Virginia, thought that, until the millenium, quackery would exist in the profession to some extent, and it was vain to legislate against it.

On motion of Dr. SAYRE, of New York, the Association took a recess of one hour, without disposing of the resolution.

*Afternoon Session.*

The Association was called to order at 1½ P. M.

The consideration of Dr. Gooch's resolution was resumed.

Dr. STILLE, of Pennsylvania, offered the following:

*Resolved,* That in order to preserve the purity and honor of the medical profession, and to place around young practitioners an additional safe-guard against temptations to do wrong, as well as to draw a more distinct line of separation between true and false physicians, it is hereby recommended to the several Medical Colleges, and such other Boards as are by law authorized to examine candidates for admission into the medical profession, to require from every graduate or licentiate, his signature to the Code of Ethics of this Association, as well as to furnish him with a copy of the Code, and it is also recommended that the formal administration of a pledge, faithfully to observe and keep the same, form part of the public ceremonies of Medical Commencement.

After some discussion the whole subject was referred to a Committee of three, to report as soon as possible.

Dr. STEWART, on behalf of Committee of Arrangements, reported the names of delegates who had registered since last report, and recommending to invite Dr. HARVEY P. PEET, Institution for Deaf and Dumb, and Dr. W. C. BUTLER, to take seats in the Association. The report suggested that when the Society adjourn it be to Saturday morning. Accompanying, was a letter from Dr. THOS. SPENCER, presenting a paper on the atomic theory of life and vital heat, as applicable to Pathology.

On motion, Dr. SPENCER made a brief extract of his views.

Dr. BLATCHFORD, of New York, moved to have a resolution, offered previously, on the licensing power, with amendments by Dr. Garnett, taken up for consideration.

Dr. HOOKER, of Connecticut, spoke to the subject of the resolutions. He would expose individual cases, without referring to the Colleges or individuals by name. The object of such an Association as this, was not to be personal, but to reform abuses. Dr. H. cited several cases, for which he stated he had the best authority, in which young men had been allowed to graduate after terms of study under one year. Four of these cases were from schools very prominent in the community. If such instances as he had stated had come without inquiry, was it not probable that if a searching inquiry were instituted numerous such abuses would be discovered? The cause of this was in a great degreee owing to the competition between schools. He wished that the subject would be brought before a committee; and he thought the remedy proposed in Dr. Garnett's amendment would prove efficacious.

Dr. JOHNSON, of St. Louis, was happy the subject had come before this Association. He was sorry the gentleman had not given the instances he referred to; for he was confident that the Association was composed of gentlemen who would have an influence in preventing such abuses. In Missouri, they had memorialized the Legislature, and found that no hope for any measure to protect the Medical Faculty could be expected from the Legislature. This Association would, in itself have a much greater influence, by denying those scholars, who acted in the manner described, a place on this floor. The moral power of this Association, if exercised, would have the effect of keeping members of the profession from such practices.

Dr. ATLEE, of Pennsylvania, rose to prevent a discussion on the subject, which could lead to no practical good for the present. The evil was a very great one, and he hoped the whole subject would be referred to a committee to make their report at the next meeting.

The subject was referred to the following Committee:

Drs. Samuel Jackson, T. Blatchford, Johnson, of Missouri, and Peaslee, of New Hampshire.

On motion of Dr. ATLEE, the subject of proposed amendments to the Constitution was taken up, and the original articles, with proposed amendments, read by the Secretary.

Dr. STEVENS, of New York, moved an indefinite postponement of the whole subject of amendments to the Constitution.

Dr. ATKINSON, of Virginia, moved to have the amendment proposing to admit four delegates from the United States Army and United States Navy, excepted from motion to postpone.

Dr. COOLIDGE, of United States Army, hoped the Association would decide whether delegates from the Army and Navy were entitled to a seat on the same footing as other delegates. If the Association did not do so, he thought the Chief of the Army and Navy Medical Bureau would not be inclined to nominate delegates from that department, in future.

Dr. BOLTON, of Virginia, read some resolutions intrusted to him by the Medical Society of Virginia, on the subject of amending the Constitution.

Dr. STEWART said the only alterations proposed were, in effect, to have one delegate from each College, and one from each hospital, instead of two. This was that they might obtain, as nearly as possible, a uniform representation of ten per cent., from all branches of the profession. Also the change respecting the Army and Navy delegates.

The question on indefinite postponement, excepting the clause altering the Constitution in relation to delegates from the Army and Navy, was put and carried, and the following adopted :

*Resolved*, That the second clause of Article 2 of the Constitution be so amended as to admit the American Medical Society in Paris to representation in this body upon the same terms as the Medical bodies in this country.

Dr. ALFRED STILLE, Chairman of the Committee to whom was referred sundry memorials touching the course to be pursued by Medical Colleges and other Boards in the examination of candidates and the granting of Diplomas reported, submitting the following resolution for adoption :

*Resolved*, That in order to preserve the purity and honor of the Medical Profession, and to place around young practitioners additional safeguards against temptations to do wrong, as well as to draw a more distinct line of separation between true and false physicians, it be and is hereby recommended, that every graduate in Medicine be required to subscribe a pledge to submit to the revocation of his diploma upon conviction of having knowingly violated the Code of Ethics of this Association. It is also recommended to the several Medical Colleges and such other Boards as are by law authorized to examine candidates for admission into the Medical Profession, to require from every graduate or licentiate his signature to the Code of Ethics of this Association, and to furnish him with a copy of the same. It is further recommended that the formal administration of a pledge faithfully to observe and keep the said Code form part of the public exercises of Medical Commencements.

The following form of Promise was among the documents referred to Committee on Pledge :

I, A. B., of ——, in the State of ———, do hereby promise, on the honor of a gentleman, that I will conform strictly with the Code of Ethics of this my Alma Mater in all things pertaining to the practice of my profession ; and when I shall fail to do so, I hereby grant to the Faculty of said School full power and authority to withdraw said Diploma, and all the rights and privileges which it is intended to confer.

Dr. PALMER and other delegates opposed that part of the report proposing to clothe Colleges with the power of revoking diplomas for a breach of the "Code of Ethics."

Several motions and counter-motions were made. The Chairman decided on the right of the Committee to withdraw the objectionable resolution, when the second and third recommendations of the report were adopted.

Dr. PALMER moved the following, which was adopted :

*Resolved*, That the Standing Committee, of which Dr. Bolton is Chairman, be instructed to inquire into all cases of death that may be reported as occurring from the use of anæsthetic agents duriag the present year in the United States, and report to the next meeting of the Association.

Dr. ZIEGLER, of Pennsylvania, moved the following, which were laid on the table:

*Resolved*, That a Committee of three or more be appointed by the President, to devise or consider some comprehensive plan or system by which subjects connected more especially with Medical Science can be more speedily, systematically, generally and thoroughly investigated and examined.

Dr. BOLTON, of Va., gave notice that he would propose the amendments to the Constitution submitted to this Association by the meeting at Richmond, last year, and which have been here indefinitely postponed, for adoption at the next annual meeting in St. Louis.

Dr. J. M. SMITH, of New York, read the following

*Report of Committee on Nominations.*

The Committee on Nominations, in fulfilling the duty of their appointment, propose to continue most of the Special Committees appointed by the Association, in May, 1851, and May, 1852, and to appoint several new Special Committees, with the subjects to them committed:

1. Dr. D. F. CONDIE, of Philadelphia, Penn.—"On the Causes of Tubercular Disease."

2. Dr. JAMES JONES, of New Orleans, La.—"On the Mutual Relations of Yellow and Bilious Remittent Fever."

3. Dr. R. S. HOLMES, of St. Louis, Mo.—"On Epidemic Erysipelas."

4. Dr. GEO. B. WOOD, of Philadelphia, Penn.—"On Diseases of Parasitic Origin."

5. Dr. R. D. ARNOLD, of Savannah, Ga.—"On the Physiological Peculiarities and Diseases of Negroes."

6. Dr. JAMES R. WOOD, of New York—"On Statistics of the Operation for the removal of Stone in the Bladder."

7. Dr. F. PEYRE PORCHER, of Charleston, S. C.—"On Toxicological and Medicinal Properties of our Cryptogamic Plants."

8. Dr. GOODRICH A. WILSON, of Virginia—"On Cholera, and its Relation to Congestive Fever—their Analogy or Identity."

9. Dr. WORTHINGTON HOOKER, of Connecticut—"On Epidemics of New England and New York."

10. Dr. JOHN L. ATLEE, of Lancaster, Penn.—"On Epidemics of New Jersey, Pennsylvania, Delaware and Maryland."

11. Dr. D. G. CAIN, of Charleston, S. C.—"On Epidemics of Mississippi, Louisiana, Texas and Arkansas."

12. Dr. W. L. SUTTON, of Georgetown, Ky.—"On Epidemics of Tennessee and Kentucky."

13. Dr. THOMAS KEYBURN, of St. Louis, Mo.—"On Epidemics of Missouri, Illinois, Iowa, and Wisconsin."

14. Dr. George Mendenhall, of Cincinnati, Ohio—"On Epidemics of Ohio, Indiana and Michigan."

15. Dr. E. D. Fenner, of New Orleans, La.—"On Epidemics of Mississippi, Louisiana, Texas and Arkansas."

16. Dr. Chas. A. Lee, of New York—"On Domestic Hygiene."

17. Dr. Daniel Brainard, of Chicago, Ill.—"On the Constitutional and Local Treatment of Carcinoma."

18. Dr. N. S. Davis, of Chicago, Ill.—"On the Influence of Local Circumstances on the Origin and Prevalence of Typhoid Fever."

19. Dr. Geo. Engelman, of St. Louis, Mo.—"On the Influence of Geological Formation on the Character of Disease."

20. Dr. Henry M. Bullitt, of Louisville, Ky.—"On the Use and Effect of Applications of Nitrate of Silver to the Throat, either in Local or General Disease."

21. Dr. Robert Campbell, of Augusta, Ga.—"On the Pathogenic Influence of Feather Beds."

22. Dr. James Bolton, of Richmond, Va.—"On the Administration of Anæsthetic Agents during Parturition."

23. Dr. Henry Taylor, of Mount Clemens, Mich.—"On Dysentery."

24. Dr. F. Donaldson, of Baltimore, Md.—"On the Present and Prospective Value of the Microscope in Disease."

25. Dr. R. L. Howard, of Columbus, Ohio—"On the Pathology and Treatment of Scrofula."

*Committee on Plans of Organization for State and County Societies.*—Isaac Hays, M. D., of Pennsylvania, Chairman; Worthington Hooker, M. D., of Connecticut; Josiah Andrews, M. D., of Michigan; B. R. Wellford, M. D., of Virginia; A. L. Pierson, M. D., of Massachusetts.

*Committee on Medical Literature.*—T. S. Bell, M D., of Kentucky, Chairman; Samuel H. Pennington, M. D., of New Jersey; Ed. H. Parker, M. D., of New Hampshire; William K. Bowling, M. D., of Tennessee; Zina Pitcher, M. D. of Michigan.

*Committee on Medical Education.*—B. R. Wellford, M. D., of Virginia, Chairman; Resign Lowe, M. D., of Iowa; Lynden A. Smith, M. D., of New Jersey; Jacob Bigelow, M. D., of Massachusetts; L. A. Dugas, M. D., of Georgia.

*Committee on Volunteer Communications*—Drs. C. A. Pope, Thos. Reyburn, John S. Moore, J. B. Johnson and A. Litton, of St. Louis, Missouri.

*Committee of Arrangements*—Drs. J. R. Washington, J. S. Moore, S. Pollock, Thos. Reyburn, J. O. Farrar, W. M. McPheeters, C. W. Hempstead and E. S. Lemoine, of St. Louis, Mo.

*Committee on Publications*—Dr. D. V. Condie, Pennsylvania, Chairman; Dr. E. L. Beadle, of New York; Dr. A. Stillé, Pennsylvania; Dr. J. Hays, Pennsylvania; Dr. E. S. Lemoine, of Missouri; Dr. G. Emerson, Pennsylvania; Dr. G. W. Norris, Pennsylvania.

Dr. Atlee, of Pennsylvania, moved that when the meeting adjourn, it adjourn to meet at 9 o'clock on Saturday morning.

The motion was withdrawn.

Dr. ROGERS, of Philadelphia, moved to have the report and resolutions offered by Dr. Zeigler, and laid on the table, taken up.

The report and resolutions were referred to a Committee, consisting of Drs. Zeigler, Rogers and Jackson.

Drs. ATLEE and MILLENBERGER offered the following, which was adopted:

*Resolved,* That the cordial thanks of the American Medical Association be, and they are hereby tendered to the Committee of Arrangement, the Trustees of the Church in which they have held their meetings, the profession and the citizens generally of New York, for the generous and elegant hospitality extended towards its members during its present session.

Dr. BOLTON, proposed a vote of thanks to the Presidents of the public Institutions, which had been thrown open for members of the Association during their stay.

Dr. BOLTON, of Virginia, moved the thanks of the Association to the Press of this City, for its accurate report of their proceedings.

On motion, it was resolved to meet on board the steamboat, at foot of Pier No. 3, this morning at 9 o'clock, and proceed to visit the public institutions belonging to the City of New York.

The President congratulated members on the close of their deliberations, and expressed his wish that they should all meet at St. Louis next year.

The Association then adjourned *sine die.*

